# 5-Bromo­spiro­[1,2-dioxane-4,4′-tricyclo­[4.3.1.1^3,8^]undeca­ne]-3′-ol

**DOI:** 10.1107/S1600536809054762

**Published:** 2009-12-24

**Authors:** Tony V. Robinson, Dennis K. Taylor, Edward R. T. Tiekink

**Affiliations:** a Discipline of Chemistry, University of Adelaide, 5005 South Australia, Australia; b Discipline of Wine and Horticulture, University of Adelaide, Waite Campus, Glen, Osmond 5064, South Australia, Australia; c Department of Chemistry, University of Malaya, 50603 Kuala Lumpur, Malaysia

## Abstract

The title compound, C_14_H_21_BrO_3_, comprises a seven- (C_7_) and three six-membered (1 × O_2_C_4_ and 2 × C_6_) rings, and each adopts a conformation based on a chair. Stability to the mol­ecular structure is afforded by an intra­molecular O—H⋯Br hydrogen bond. In the crystal structure, mol­ecules are arranged into a helical supra­molecular chain along the *b* axis, linked by C—H⋯O inter­actions, where the O-atom acceptor is one of the dioxane O atoms. The crystal studied was found to be a racemic twin. The major component was present 94% of the time.

## Related literature

For the background to endoperoxides, see: Casteel (1999 ▶); Tang *et al.* (2004 ▶). For the potential of simple 1,2-dioxines and ep­oxy-1,2-dioxanes as novel anti­malarial and anti­fungal agents, see: Taylor *et al.* (2004 ▶); Crespo *et al.* (2008 ▶); Macreadie *et al.* (2006 ▶, 2008 ▶); Avery *et al.* (2007 ▶). For the synthesis of related compounds, see: Robinson (2003 ▶).
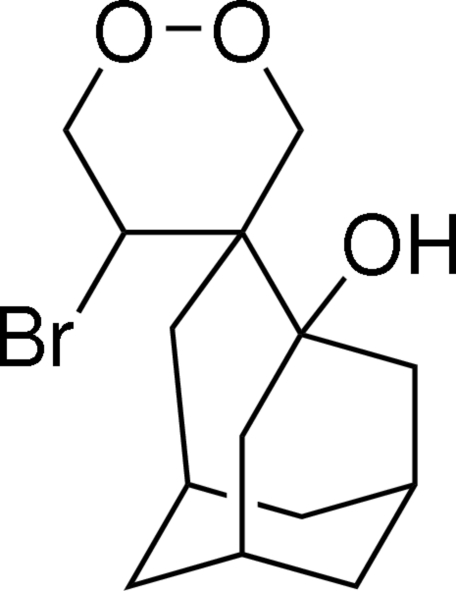


## Experimental

### 

#### Crystal data

C_14_H_21_BrO_3_
*M* *_r_* = 317.22
Monoclinic, 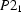

*a* = 8.6199 (7) Å
*b* = 6.6370 (5) Å
*c* = 11.7171 (9) Å
β = 105.426 (2)°
*V* = 646.19 (9) Å^3^
*Z* = 2
Mo *K*α radiation
μ = 3.18 mm^−1^
*T* = 293 K
0.19 × 0.11 × 0.08 mm

#### Data collection

Bruker SMART CCD diffractometer
Absorption correction: multi-scan (*SADABS*; Bruker, 2000 ▶) *T*_min_ = 0.657, *T*_max_ = 1
4618 measured reflections
2140 independent reflections
2063 reflections with *I* > 2σ(*I*)
*R*_int_ = 0.040

#### Refinement

*R*[*F* ^2^ > 2σ(*F* ^2^)] = 0.034
*wR*(*F* ^2^) = 0.094
*S* = 1.04
2140 reflections
167 parameters
2 restraints
H-atom parameters constrained
Δρ_max_ = 0.77 e Å^−3^
Δρ_min_ = −0.49 e Å^−3^

### 

Data collection: *SMART* (Bruker, 2000 ▶); cell refinement: *SAINT* (Bruker, 2000 ▶); data reduction: *SAINT*; program(s) used to solve structure: *PATTY* in *DIRDIF92* (Beurskens *et al.*, 1992 ▶); program(s) used to refine structure: *SHELXL97* (Sheldrick, 2008 ▶); molecular graphics: *DIAMOND* (Brandenburg, 2006 ▶); software used to prepare material for publication: *publCIF* (Westrip, 2010 ▶).

## Supplementary Material

Crystal structure: contains datablocks global, I. DOI: 10.1107/S1600536809054762/hb5281sup1.cif

Structure factors: contains datablocks I. DOI: 10.1107/S1600536809054762/hb5281Isup2.hkl

Additional supplementary materials:  crystallographic information; 3D view; checkCIF report

## Figures and Tables

**Table 1:** Hydrogen-bond geometry (Å, °)

*D*—H⋯*A*	*D*—H	H⋯*A*	*D*⋯*A*	*D*—H⋯*A*
O3—H3*o*⋯Br	0.84	2.36	3.128 (3)	153
C2—H2a⋯O1^i^	0.98	2.59	3.560 (4)	171
C14—H14b⋯O1^i^	0.98	2.56	3.513 (4)	165

Symmetry code: (i) 
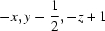
.

**Fig. 1.**
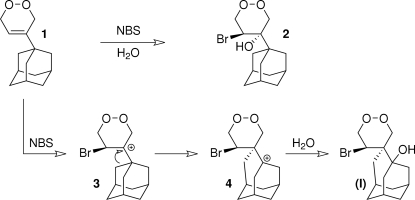
Reaction scheme.

**Fig. 2.**
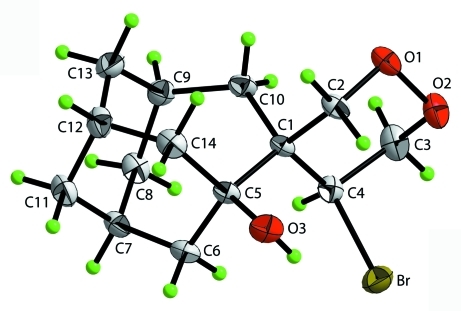
Molecular structure of (I) showing atom-labelling scheme and displacement ellipsoids at the 50% probability level.

**Fig. 3.**
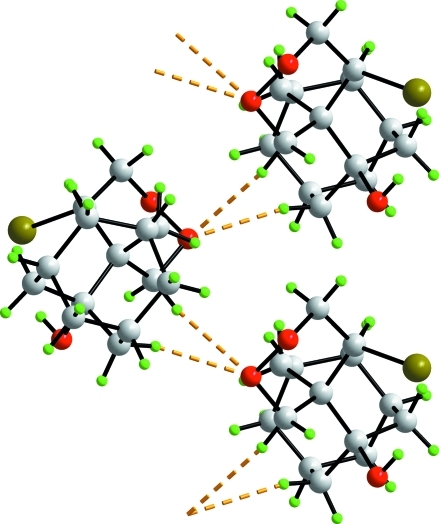
Supramolecular chain formation along the b axis in (I) mediated by C—H···O interactions (orange dashed lines).

## supplementary crystallographic information

### Comment

Endoperoxides comprise a diverse range of compounds often displaying interesting biological activities (Casteel, 1999; Tang *et al.*, 2004). Recently, we investigated the potential of simple 1,2-dioxines and epoxy-1,2-dioxanes as novel antimalarial (Taylor *et al.*, 2004; Crespo *et al.*, 2008) and antifungal (Macreadie *et al.*, 2006; Avery *et al.*, 2007; Macreadie *et al.*, 2008) agents. During the course of these studies other modified 1,2-dioxines were prepared, particularly by electrophilic additions to the alkene unit, which included halo-hydrins (Robinson, 2003). The title compound (I) was obtained as a minor product from the attempted bromo-hydrin addition to compound 1, presumably through carbocation migration (Fig. 1).

The molecular structure of (I), Fig. 2, features a close intramolecular O–H···Br hydrogen bond as both substituents lie to the same side of the molecule, Table 1. This interaction closes a six-membered {···HOC_3_Br} ring with a half-chair conformation. The six-membered O_2_C_4_ ring has a twisted chair conformation and the bromide occupies an axial position. The two six-membered C_6_ rings share the C7, C11, and C12 atoms, and each has a slightly twisted chair conformation. The hydroxyl group occupies a bisectional position relative to the ring to which it is connected. Finally, the seven-membered ring comprising the C1,C5–C10 atoms has a distorted chair conformation with the C5 and C8 atoms occupying positions above and below the plane defined by the remaining five atoms. In the crystal structure, the primary intermolecular interactions are of the type C—H···O, Table 1. The dioxane-O1 atom forms two close C—H···O contacts to form a supramolecular helical chain aligned along [010], Fig. 3 and Table 1.

### Experimental

Referring to the reaction scheme shown in Fig. 1, to a stirred solution of 1 (200 mg, 0.91 mmol) in acetone (5 ml) was added water (180 mg, 10 mmol) and *N*-bromosuccinimde (219 mg, 1.23 mmol). The mixture was stirred at ambient temperature until TLC indicated complete consumption of starting material (*ca* 3 h). The reaction was then diluted with CH_2_Cl_2_ (50 ml), washed with sat. NaHCO_3_ solution (2 *x* 20 ml), and dried (Na_2_SO_4_). The solvent was removed *in vacuo* yielding a crude multi-component mixture which was purified by flash chromatography eluting with 3:1 CH_2_Cl_2_/hexane. Fractions of interest (*R_f_* 0.20 in 3:1 CH_2_Cl_2_/hexane) were combined and concentrated giving a solid white residue, which was recrystallized from a slowly evaporating a 1:1 mixture of dichloromethane/heptane producing the title compound (I) as colourless needles.

### Refinement

Carbon-bound H-atoms were placed in calculated positions (C–H 0.98–0.99 Å) and were included in the refinement in the riding model approximation with *U*_iso_(H) set to 1.2*U*_eq_(C). The O–bound H-atom was located in a difference Fourier map and was refined with an O–H restraint of 0.840±0.001 Å, and with *U_iso_*(H) = 1.*5U_eq_*(O). The structure was refined as a racemic twin precluding the determination of the absolute structure.

### Crystal data

**Table:**

C_14_H_21_BrO_3_	*F*(000) = 328
*M**_r_* = 317.22	*D*_x_ = 1.630 Mg m^−^^3^
Monoclinic, *P*2_1_	Mo *K*α radiation, λ = 0.71069 Å
Hall symbol: P 2yb	Cell parameters from 2705 reflections
*a* = 8.6199 (7) Å	θ = 2.5–29.5°
*b* = 6.6370 (5) Å	µ = 3.18 mm^−^^1^
*c* = 11.7171 (9) Å	*T* = 293 K
β = 105.426 (2)°	Block, colourless
*V* = 646.19 (9) Å^3^	0.19 × 0.11 × 0.08 mm
*Z* = 2	

### Data collection

**Table:**

Bruker SMART CCD diffractometer	2140 independent reflections
Radiation source: fine-focus sealed tube	2063 reflections with *I* > 2σ(*I*)
graphite	*R*_int_ = 0.040
ω scans	θ_max_ = 27.5°, θ_min_ = 1.8°
Absorption correction: multi-scan (*SADABS*; Bruker, 2000)	*h* = −11→11
*T*_min_ = 0.657, *T*_max_ = 1	*k* = −5→8
4618 measured reflections	*l* = −14→15

### Refinement

**Table:**

Refinement on *F*^2^	Primary atom site location: structure-invariant direct methods
Least-squares matrix: full	Secondary atom site location: difference Fourier map
*R*[*F*^2^ > 2σ(*F*^2^)] = 0.034	Hydrogen site location: inferred from neighbouring sites
*wR*(*F*^2^) = 0.094	H-atom parameters constrained
*S* = 1.04	*w* = 1/[σ^2^(*F*_o_^2^) + (0.0714*P*)^2^ + 0.0296*P*] where *P* = (*F*_o_^2^ + 2*F*_c_^2^)/3
2140 reflections	(Δ/σ)_max_ = 0.001
167 parameters	Δρ_max_ = 0.77 e Å^−^^3^
2 restraints	Δρ_min_ = −0.49 e Å^−^^3^

### Special details

**Table:**

Geometry. All s.u.'s (except the s.u. in the dihedral angle between two l.s. planes) are estimated using the full covariance matrix. The cell s.u.'s are taken into account individually in the estimation of s.u.'s in distances, angles and torsion angles; correlations between s.u.'s in cell parameters are only used when they are defined by crystal symmetry. An approximate (isotropic) treatment of cell s.u.'s is used for estimating s.u.'s involving l.s. planes.
Refinement. Refinement of *F*^2^ against ALL reflections. The weighted *R*-factor *wR* and goodness of fit *S* are based on *F*^2^, conventional *R*-factors *R* are based on *F*, with *F* set to zero for negative *F*^2^. The threshold expression of *F*^2^ > 2σ(*F*^2^) is used only for calculating *R*-factors(gt) *etc*. and is not relevant to the choice of reflections for refinement. *R*-factors based on *F*^2^ are statistically about twice as large as those based on *F*, and *R*- factors based on ALL data will be even larger.

### Fractional atomic coordinates and isotropic or equivalent isotropic displacement parameters (Å^2^)

**Table:**

	*x*	*y*	*z*	*U*_iso_*/*U*_eq_	
Br	0.03137 (4)	0.40212 (10)	0.92113 (2)	0.03533 (13)	
O1	−0.0876 (3)	0.3711 (5)	0.5673 (2)	0.0309 (6)	
O2	−0.1461 (3)	0.5051 (5)	0.6461 (3)	0.0346 (6)	
O3	0.1635 (3)	0.0061 (4)	0.8367 (2)	0.0295 (6)	
H3o	0.1331	0.0876	0.8811	0.044*	
C1	0.1666 (4)	0.3109 (5)	0.7167 (2)	0.0183 (6)	
C2	0.0084 (4)	0.2208 (5)	0.6406 (3)	0.0241 (7)	
H2A	0.0326	0.1130	0.5908	0.029*	
H2B	−0.0521	0.1619	0.6923	0.029*	
C3	−0.0120 (4)	0.6195 (6)	0.7095 (4)	0.0313 (8)	
H3A	−0.0476	0.7184	0.7593	0.038*	
H3B	0.0332	0.6933	0.6534	0.038*	
C4	0.1193 (4)	0.4853 (5)	0.7878 (3)	0.0227 (6)	
H4	0.2159	0.5693	0.8202	0.027*	
C5	0.2688 (4)	0.1354 (5)	0.7913 (3)	0.0206 (6)	
C6	0.4047 (4)	0.2092 (6)	0.8964 (3)	0.0254 (7)	
H6A	0.3659	0.3262	0.9317	0.031*	
H6B	0.4291	0.1026	0.9564	0.031*	
C7	0.5598 (4)	0.2675 (6)	0.8661 (3)	0.0259 (7)	
H7	0.6410	0.3026	0.9406	0.031*	
C8	0.5395 (4)	0.4492 (5)	0.7823 (3)	0.0270 (7)	
H8A	0.6464	0.4966	0.7801	0.032*	
H8B	0.4874	0.5584	0.8145	0.032*	
C9	0.4400 (3)	0.4049 (7)	0.6548 (2)	0.0257 (6)	
H9	0.4676	0.5136	0.6057	0.031*	
C10	0.2557 (4)	0.4135 (7)	0.6318 (2)	0.0241 (6)	
H10A	0.2098	0.3566	0.5528	0.029*	
H10B	0.2258	0.5563	0.6272	0.029*	
C11	0.6215 (4)	0.0862 (7)	0.8115 (3)	0.0328 (8)	
H11A	0.6336	−0.0298	0.8649	0.039*	
H11B	0.7268	0.1167	0.7987	0.039*	
C12	0.5010 (4)	0.0371 (6)	0.6937 (3)	0.0296 (7)	
H12	0.5392	−0.0842	0.6601	0.035*	
C13	0.4948 (5)	0.2104 (7)	0.6087 (4)	0.0342 (9)	
H13A	0.4205	0.1768	0.5321	0.041*	
H13B	0.6019	0.2305	0.5965	0.041*	
C14	0.3364 (4)	−0.0107 (6)	0.7144 (3)	0.0264 (7)	
H14A	0.3432	−0.1445	0.7508	0.032*	
H14B	0.2578	−0.0196	0.6369	0.032*	

### Atomic displacement parameters (Å^2^)

**Table:**

	*U*^11^	*U*^22^	*U*^33^	*U*^12^	*U*^13^	*U*^23^
Br	0.03383 (19)	0.0473 (2)	0.02721 (17)	−0.00431 (19)	0.01216 (12)	−0.00797 (18)
O1	0.0285 (10)	0.0314 (16)	0.0266 (10)	0.0015 (13)	−0.0033 (8)	0.0014 (11)
O2	0.0235 (12)	0.0337 (15)	0.0430 (15)	0.0023 (11)	0.0027 (11)	−0.0026 (12)
O3	0.0362 (14)	0.0220 (13)	0.0307 (13)	−0.0085 (11)	0.0096 (11)	0.0070 (10)
C1	0.0191 (13)	0.0172 (14)	0.0163 (12)	−0.0015 (12)	0.0005 (11)	0.0006 (11)
C2	0.0251 (15)	0.0206 (16)	0.0221 (15)	−0.0037 (14)	−0.0015 (12)	−0.0012 (12)
C3	0.0270 (17)	0.0204 (17)	0.0436 (19)	−0.0016 (14)	0.0046 (15)	−0.0023 (15)
C4	0.0210 (14)	0.0211 (14)	0.0253 (15)	−0.0039 (13)	0.0047 (12)	−0.0030 (12)
C5	0.0238 (14)	0.0177 (15)	0.0189 (13)	−0.0021 (13)	0.0029 (11)	0.0014 (11)
C6	0.0296 (16)	0.0264 (17)	0.0171 (13)	0.0002 (14)	0.0005 (12)	0.0018 (12)
C7	0.0252 (15)	0.0264 (18)	0.0218 (14)	0.0012 (14)	−0.0013 (12)	−0.0012 (13)
C8	0.0248 (14)	0.0221 (18)	0.0318 (15)	−0.0052 (12)	0.0035 (12)	−0.0022 (13)
C9	0.0252 (13)	0.0274 (15)	0.0249 (12)	−0.005 (2)	0.0075 (10)	0.0022 (19)
C10	0.0275 (13)	0.0235 (15)	0.0204 (11)	−0.0014 (17)	0.0051 (10)	0.0058 (15)
C11	0.0257 (16)	0.032 (2)	0.0355 (18)	0.0069 (15)	−0.0003 (14)	−0.0029 (16)
C12	0.0258 (16)	0.0273 (17)	0.0339 (17)	0.0035 (14)	0.0049 (14)	−0.0106 (15)
C13	0.0317 (18)	0.047 (2)	0.0264 (15)	−0.0014 (17)	0.0117 (13)	−0.0060 (16)
C14	0.0318 (16)	0.0157 (14)	0.0285 (16)	−0.0014 (14)	0.0024 (13)	−0.0049 (13)

### Geometric parameters (Å, °)

**Table:**

Br—C4	1.987 (3)	C7—C11	1.524 (5)
O1—C2	1.428 (4)	C7—C8	1.535 (5)
O1—O2	1.465 (4)	C7—H7	0.9900
O2—C3	1.417 (5)	C8—C9	1.540 (4)
O3—C5	1.449 (4)	C8—H8A	0.9800
O3—H3O	0.8400	C8—H8B	0.9800
C1—C2	1.538 (4)	C9—C13	1.522 (6)
C1—C4	1.543 (5)	C9—C10	1.540 (4)
C1—C10	1.566 (4)	C9—H9	0.9900
C1—C5	1.577 (4)	C10—H10A	0.9800
C2—H2A	0.9800	C10—H10B	0.9800
C2—H2B	0.9800	C11—C12	1.526 (5)
C3—C4	1.537 (5)	C11—H11A	0.9800
C3—H3A	0.9800	C11—H11B	0.9800
C3—H3B	0.9800	C12—C13	1.513 (6)
C4—H4	0.9900	C12—C14	1.535 (5)
C5—C6	1.537 (4)	C12—H12	0.9900
C5—C14	1.539 (5)	C13—H13A	0.9800
C6—C7	1.522 (5)	C13—H13B	0.9800
C6—H6A	0.9800	C14—H14A	0.9800
C6—H6B	0.9800	C14—H14B	0.9800
			
C2—O1—O2	106.6 (2)	C8—C7—H7	108.2
C3—O2—O1	106.6 (3)	C7—C8—C9	114.2 (3)
C5—O3—H3O	100.1	C7—C8—H8A	108.7
C2—C1—C4	106.5 (3)	C9—C8—H8A	108.7
C2—C1—C10	108.0 (2)	C7—C8—H8B	108.7
C4—C1—C10	105.2 (3)	C9—C8—H8B	108.7
C2—C1—C5	108.2 (3)	H8A—C8—H8B	107.6
C4—C1—C5	116.4 (2)	C13—C9—C8	111.2 (3)
C10—C1—C5	112.2 (3)	C13—C9—C10	111.8 (3)
O1—C2—C1	111.1 (3)	C8—C9—C10	116.4 (3)
O1—C2—H2A	109.4	C13—C9—H9	105.5
C1—C2—H2A	109.4	C8—C9—H9	105.5
O1—C2—H2B	109.4	C10—C9—H9	105.5
C1—C2—H2B	109.4	C9—C10—C1	122.0 (3)
H2A—C2—H2B	108.0	C9—C10—H10A	106.8
O2—C3—C4	111.8 (3)	C1—C10—H10A	106.8
O2—C3—H3A	109.3	C9—C10—H10B	106.8
C4—C3—H3A	109.3	C1—C10—H10B	106.8
O2—C3—H3B	109.3	H10A—C10—H10B	106.7
C4—C3—H3B	109.3	C7—C11—C12	108.6 (3)
H3A—C3—H3B	107.9	C7—C11—H11A	110.0
C3—C4—C1	111.8 (3)	C12—C11—H11A	110.0
C3—C4—Br	104.8 (2)	C7—C11—H11B	110.0
C1—C4—Br	115.2 (2)	C12—C11—H11B	110.0
C3—C4—H4	108.3	H11A—C11—H11B	108.4
C1—C4—H4	108.3	C13—C12—C11	109.2 (3)
Br—C4—H4	108.3	C13—C12—C14	112.9 (3)
O3—C5—C6	108.2 (3)	C11—C12—C14	109.6 (3)
O3—C5—C14	102.3 (3)	C13—C12—H12	108.3
C6—C5—C14	110.1 (3)	C11—C12—H12	108.3
O3—C5—C1	109.2 (2)	C14—C12—H12	108.3
C6—C5—C1	113.8 (3)	C12—C13—C9	111.8 (3)
C14—C5—C1	112.6 (2)	C12—C13—H13A	109.3
C7—C6—C5	115.1 (3)	C9—C13—H13A	109.3
C7—C6—H6A	108.5	C12—C13—H13B	109.3
C5—C6—H6A	108.5	C9—C13—H13B	109.3
C7—C6—H6B	108.5	H13A—C13—H13B	107.9
C5—C6—H6B	108.5	C12—C14—C5	118.2 (3)
H6A—C6—H6B	107.5	C12—C14—H14A	107.8
C6—C7—C11	108.9 (3)	C5—C14—H14A	107.8
C6—C7—C8	113.0 (3)	C12—C14—H14B	107.8
C11—C7—C8	110.2 (3)	C5—C14—H14B	107.8
C6—C7—H7	108.2	H14A—C14—H14B	107.1
C11—C7—H7	108.2		
			
C2—O1—O2—C3	71.9 (3)	C1—C5—C6—C7	−83.6 (4)
O2—O1—C2—C1	−70.2 (3)	C5—C6—C7—C11	−58.4 (4)
C4—C1—C2—O1	56.4 (3)	C5—C6—C7—C8	64.3 (4)
C10—C1—C2—O1	−56.1 (4)	C6—C7—C8—C9	−70.6 (4)
C5—C1—C2—O1	−177.8 (3)	C11—C7—C8—C9	51.5 (4)
O1—O2—C3—C4	−63.0 (4)	C7—C8—C9—C13	−47.0 (4)
O2—C3—C4—C1	52.4 (4)	C7—C8—C9—C10	82.7 (4)
O2—C3—C4—Br	−73.1 (3)	C13—C9—C10—C1	83.5 (4)
C2—C1—C4—C3	−45.5 (3)	C8—C9—C10—C1	−45.8 (6)
C10—C1—C4—C3	69.0 (3)	C2—C1—C10—C9	−142.2 (4)
C5—C1—C4—C3	−166.2 (3)	C4—C1—C10—C9	104.4 (4)
C2—C1—C4—Br	74.1 (3)	C5—C1—C10—C9	−23.0 (5)
C10—C1—C4—Br	−171.44 (19)	C6—C7—C11—C12	65.3 (4)
C5—C1—C4—Br	−46.6 (3)	C8—C7—C11—C12	−59.2 (4)
C2—C1—C5—O3	−42.1 (3)	C7—C11—C12—C13	64.5 (4)
C4—C1—C5—O3	77.7 (3)	C7—C11—C12—C14	−59.6 (4)
C10—C1—C5—O3	−161.2 (3)	C11—C12—C13—C9	−61.1 (4)
C2—C1—C5—C6	−163.1 (3)	C14—C12—C13—C9	61.2 (4)
C4—C1—C5—C6	−43.4 (4)	C8—C9—C13—C12	51.4 (4)
C10—C1—C5—C6	77.8 (4)	C10—C9—C13—C12	−80.6 (4)
C2—C1—C5—C14	70.8 (3)	C13—C12—C14—C5	−73.5 (4)
C4—C1—C5—C14	−169.5 (3)	C11—C12—C14—C5	48.5 (4)
C10—C1—C5—C14	−48.3 (3)	O3—C5—C14—C12	−154.2 (3)
O3—C5—C6—C7	154.8 (3)	C6—C5—C14—C12	−39.3 (4)
C14—C5—C6—C7	43.8 (4)	C1—C5—C14—C12	88.7 (4)

### Hydrogen-bond geometry (Å, °)

**Table:**

*D*—H···*A*	*D*—H	H···*A*	*D*···*A*	*D*—H···*A*
O3—H3o···Br	0.84	2.36	3.128 (3)	153
C2—H2a···O1^i^	0.98	2.59	3.560 (4)	171
C14—H14b···O1^i^	0.98	2.56	3.513 (4)	165

Symmetry codes:  (i) −*x*, *y*−1/2, −*z*+1.
